# The Utilization of the Immune System in Lung Cancer Treatment: Beyond Chemotherapy

**DOI:** 10.3390/ijms17030286

**Published:** 2016-02-25

**Authors:** Carmen W. H. Chan, Stephen K. W. Tsui, Bernard M. H. Law, Winnie K. W. So, Fiona W. K. Tang, Cho-Lee Wong

**Affiliations:** 1 The Nethersole School of Nursing, The Chinese University of Hong Kong, Shatin, the New Territories, Hong Kong, China; whchan@cuhk.edu.hk (C.W.H.C.); winnieso@cuhk.edu.hk (W.K.W.S.); wktang@cuhk.edu.hk (F.W.K.T.); jojowong@cuhk.edu.hk (C.-L.W.); 2 School of Biomedical Sciences, The Chinese University of Hong Kong, Shatin, the New Territories, Hong Kong, China; kwtsui@cuhk.edu.hk

**Keywords:** lung cancer, chemotherapy, immunotherapy, immune system

## Abstract

Lung cancer is ranked first worldwide as one of the main cancers in terms of prevalence and mortality rate. The development of effective treatment strategies against lung cancer is therefore of paramount importance. Traditionally, chemotherapy was employed in the treatment of various cancers. However, the non-specific nature of the actions of chemotherapeutic drugs and the potential for tumors to develop resistance to these drugs may render chemotherapy a less favorable option for cancer treatment. Immunotherapy provides an alternative strategy for this purpose. It involves the utilization of the immune system and the immune effector cells to elicit an immune response to the tumors, thereby eliminating them. Strategies include the administration of pro-inflammatory cytokines for immune stimulation, the removal of immunological checkpoints using monoclonal antibodies, and the use of cancer vaccines to enhance immunity against tumors. This article summarizes the above strategies, highlights the reasons why immunotherapy is superior to chemotherapy for the purpose of tumor removal, and reviews the recent clinical studies comparing the clinical outcomes of patients undergoing immunotherapy and chemotherapy. The article also describes advances in immunotherapeutic strategies for the treatment of lung cancer.

## 1. Introduction

Lung cancer has long been considered one of the most prevalent cancers in the world. It has become an increasing burden on human health since the 1930s, when both the incidence and mortality rates of lung cancer began rising steadily [1]. To this day, lung cancer still presents a health concern to the world population, and resulted in more than 1.6 million deaths worldwide in 2012 [2]. Therefore, the development of effective treatment strategies to counter the high global mortality rate of lung cancer is of paramount importance.

Through years of research, we have been able to gain a better understanding of the molecular mechanisms and pathways responsible for tumor formation and development. All these endeavors have contributed to the development of chemotherapy where these molecular pathways are targeted to halt tumor growth with the use of anti-cancer drugs. In addition, immunotherapy, the exploitation of the human immune system in the fight against tumors, has also emerged as an effective strategy in cancer treatment, resulting in an increased interest expressed by pharmaceutical companies in the development of immunotherapy drugs. Therefore, one may wonder why the research journey for cancer treatment should go beyond the development of chemotherapy, and why immunotherapy is seen as a milestone for cancer treatment so that it is able to attract the attention of pharmaceutical companies. This article will highlight the rationale and advantages of immunotherapy and will briefly describe the advances in immunotherapy that contribute to its success in lung cancer treatment.

## 2. Cancer Treatment—Why Should We Go Beyond Chemotherapy?

### 2.1. Mechanistic Aspects of Tumor Removal by Chemotherapeutic Drugs

Chemotherapy can be regarded as one of a series of traditional methods for cancer treatment, with its first trial dating back to 1942, when nitrogen mustard was used to treat a patient with lymphoma [3]. It involves the utilization of anti-cancer drugs as an external “tool” for removing the tumor. Sometimes, combination chemotherapy, the simultaneous utilization of more than one anti-cancer drug, may be employed in order to enhance the effectiveness of cancer treatment. To date, a repertoire of chemotherapeutic drugs has been developed, most of which target either the DNA in cells, or the components responsible for the progression of the cell cycle. For example, alkylating agents such as temozolomide and altretamine are able to add an alkyl group to the nitrogenous bases in the DNA, thereby damaging it. This prevents the expression of genes required for the cells to survive and proliferate, such as cyclins and cyclin-dependent kinases. Other chemotherapeutic drugs such as doxorubicin are able to inhibit topoisomerase, an enzyme responsible for DNA replication and therefore cell proliferation. In other words, such drugs can introduce interference to the progression of the cell cycle and thereby prevent tumor cells from proliferating.

### 2.2. Drawbacks of Chemotherapy

Despite the potential of chemotherapeutic drugs in tumor removal, it is the way in which they help remove tumors that creates problems with their use, leading to the development of adverse side effects. After a chemotherapeutic drug is administered to the body, the drug travels around the body in the bloodstream, conferring toxicity to actively proliferating cells. However, this non-specific removal of proliferating cells would also make healthy cells that are dividing be the target of the chemotherapeutic drug. Such non-specific cytotoxicity of the drugs could lead to massive cell death. This would warrant an extensive repair mechanism, causing a significant depletion of energy for individuals using the drugs. This is likely the reason why patients receiving chemotherapy experience severe fatigue. In addition, cancer patients receiving chemotherapy are also vulnerable to the damage of their vital organs. Cisplatin, a drug used in non-small cell lung cancer (NSCLC) treatment, was found to lead to kidney damage, an effect partly attributed to the production of pro-inflammatory cytokines [4]. Chemotherapy drugs were also suspected of leading to free-radical-induced hepatotoxicity, leading to liver damage [5]. Overall, the damage of healthy cells caused by chemotherapeutic drugs may cause a repertoire of adverse side effects including organ damage.

In light of such drawback, molecular oncologists had developed a more effective way of chemotherapeutic drug delivery to the tumors, so that the drug can be targeted to the tumors more specifically. The rationale of such drug delivery method is based on the phenomenon that cancer cells normally display specific antigens on their cell surface which are not present on healthy cells. Therefore, certain monoclonal antibodies that possess the ability to bind specifically to these tumor antigens can be utilized to direct the chemotherapeutic drugs specifically to the tumors. These antibodies can be conjugated to the drug to form an immuno-conjugate. It can then be administered to the body of the patients, and the antibodies would direct the drug towards the tumor, where the drug can exert its effects. Such techniques, often called targeted chemotherapy, would ensure that only tumor cells would be targeted for removal, thereby limiting the side effects caused by inappropriate removal of healthy cells.

Another major drawback of chemotherapy is the tendency of tumors to develop a resistance to chemotherapeutic drugs, increasing the potential of tumors to reappear after patients receive chemotherapy [6]. Tumor resistance is mainly caused by the increased expression of energy-dependent efflux pumps in tumors, which may prevent the drug from entering the tumor cells. Indeed, cancer cells were found to express an elevated level of Patched, a Hedgehog receptor present on the cell surface which plays a role in drug efflux [7]. This leads to an increase in the level of drug efflux and a subsequent reduction of the intake of the drug into the tumor cells, therefore limiting the access of the drug to the DNA and the proteins involved in cell cycle progression. This in turn prevents the tumors from being affected by the administered drug, rendering the drug ineffective in tumor removal. Therefore, while targeted chemotherapy may be utilized to specifically remove tumor cells, the tendency of tumors to develop resistance to chemotherapeutic drugs would warrant a search for a more effective way of cancer treatment.

## 3. Immunotherapy—Harnessing the Immune System to Remove Tumors

As discussed, chemotherapy suffers from drawbacks such as non-specificity and tumor resistance, leading to unwanted side effects and ineffectiveness in halting tumor growth respectively. In response to this, the utilization of the natural body defense system—a system that would ensure that only specific cells would be targeted for removal and that unhealthy cells would be effectively eliminated—should therefore be considered to increase the effectiveness of halting tumor progression. Such consideration has led to the development of immunotherapy, a type of cancer therapy involving the boosting of the ability of the body’s immune system in tumor removal, as an alternative strategy in cancer treatment.

In fact, the first trial concerning the use of the immune system in tumor control dates back to 1891, when William Coley discovered that an immune response against a tumor was generated following the injection of streptococcal bacteria into the tumor, thereby causing a reduction of its size [8]. However, the significance of this finding attracted little attention and interest at the time. It was not until the 1960s when studies on the utilization of T lymphocyte-mediated immune responses in tumor rejection began [9], that scientists started to realize the importance of the immune system in tumor surveillance and removal. In later years, with a better understanding of how the immune system functions in the eradication of pathogens and tumors, scientists were able to develop further novel ways to harness the immune system to assist in tumor removal. The mechanisms in which an immune response is elicited against a tumor have been previously described; in short, macrophages, a component of the immune system, will increase the production of cytokines such as tumor necrosis factor alpha (TNF-α) in the presence of tumors [10]. This phenomenon can lead to the increased ability of antigen presenting cells such as dendritic cells to present the tumor antigens to immune effector cells such as T cells. The specific binding of the antigens to the T cell receptor (TCR), together with the binding of a further molecule on the dendritic cell to the TCR that acts as a co-stimulatory signal, would result in the activation of T cells. This contributes to the activation of an immune response leading to the removal of the tumor through the cytotoxic function of T cells [11]. In this way, only the tumor containing cells displaying tumor antigens would be targeted for removal by the immune system, while none of the other healthy cells would be affected.

As discussed above, cytokines are one of the first components to be involved in an immune response against tumors, through the induction of the cytotoxic function of T cells. Therefore, cytokines are widely used in current immunotherapies. For example, combinatorial treatment of low doses of interleukin-2 (IL-2) and TNF-α in NSCLC patients has resulted in enhanced tumor regression [12], making these pro-inflammatory cytokines an attractive choice of therapeutic agent to be used in immunotherapy to treat cancer. Furthermore, immunotherapies can be combined with the use of chemotherapeutic drugs to enhance effectiveness in cancer treatment. In fact, a number of recent studies had compared the use of a combination of immunotherapeutic and chemotherapeutic drugs and the sole use of chemotherapeutic drugs on NSCLC patients. These studies are summarized in Table 1. Most of these studies show that patients undergoing combined therapies exhibited prolonged overall survival. Overall, the idea of utilizing the immune system in cancer treatment would confer the benefit of achieving specificity in tumor removal. Furthermore, the effectiveness of the process of tumor removal can be ensured as an immune response would be mounted when tumor antigens are presented.

However, there are occasions when the immune system may fail to detect the tumor and destine it for elimination, as tumors themselves may employ defense mechanisms so that they are able to escape immune surveillance. In fact, the immune system would impose certain so called “immunological checkpoints” for itself, so that it would not be able to eliminate the “self” and healthy cells during an immune response to a pathogen or tumor. For example, cytotoxic T-lymphocyte-associated protein 4 (CTLA-4) expressed on T cells, once bound to B7/cluster of differentiation 80 (CD80) molecule on the antigen-presenting cells, would elicit an inhibitory signal to compete with the immune co-stimulatory signal exhibited by the activated co-stimulatory receptor known as CD28. This keeps the activation of T cells in check, and thereby induces immune tolerance. Tumors exploit this immunological checkpoint pathway in order to prevent themselves from being detected and eliminated by the immune system. They express a constitutively low level of B7/CD80 molecule [13], resulting in an insufficient co-stimulatory signal for the T cells to become activated. This results in a failure in mounting an immune response to the tumor. This presents a barrier to the effectiveness of immunotherapy, as immunotherapy is reliant on the surveillance function of the immune system in identifying and removing cells expressing tumor antigens. To counteract this, monoclonal antibodies which target the immune inhibitory signals were developed. These antibodies can compete with the molecules conferring inhibitory signals expressed on T cells for binding to the B7/CD80 molecule. This blocks any immune inhibitory signals and ensures that the co-stimulatory signal is strong enough to elicit immune responses against tumors. For example, ipilimumab, a monoclonal antibody against CTLA-4 that was shown to improve the progression-free survival of patients with NSCLC in a randomized phase II clinical trial [14], can impede the binding of CD80 to CTLA-4 on T cells. Furthermore, nivolumab, a monoclonal antibody against programmed death-1 (PD-1) which was shown to increase the survival rates in previously treated NSCLC patients [15], is able to prevent the interaction between PD-1 on activated T cells and PD-1 ligand on tumor cells. The prevention of these molecular interactions enables the shutdown of the inhibitory signal for T cell activation, thereby preventing immune resistance of the tumor.

The effectiveness of immune checkpoint inhibitors in the treatment of NSCLC was previously documented. Indeed, Borghaei *et al.* [15] and Brahmer *et al.* [22] compared the use of nivolumab and the chemotherapeutic drug docetaxel on the clinical outcomes of NSCLC patients in phase III clinical trials, where the benefits of nivolumab on NSCLC patient survival over docetaxel were demonstrated. Such a comparison also highlights the benefits of immunotherapy in cancer treatment over chemotherapy. The findings of this study are presented in Table 2. Both the overall survival rate and one-year progression-free survival rate (PFS) were reported to be higher among patients treated with nivolumab. Further, the reported frequency of grade 3 or 4 adverse events in the group of patients treated with nivolumab was significantly lower than that treated with docetaxel, indicating a low toxicity profile of nivolumab. The benefits of nivolumab in improved patient survival, together with its low toxicity profile, led to the approval of the use of this anti-PD-1 antibody in lung cancer treatment by the U.S. Food and Drug Administration in March 2015.

Despite the apparent superiority of immunotherapy over traditional chemotherapy in terms of effectiveness in resisting tumor growth, the toxicity profiles of certain immunotherapeutic agents did raise concerns over their utilization in cancer treatment. These concerns are largely caused by the occurrence of immune-related adverse events, which were reported in previous immunotherapeutic clinical trials. For example, the use of BMS-936558, an anti-PD-1 antibody, in a clinical trial resulted in 14% of patients suffering from grade 3 or 4 adverse events. These events include rashes, fatigue and diarrhea [23]. Pembrolizumab, another anti-PD-1 antibody, was also found to cause adverse events such as fatigue, itching and rashes, although most of these events only belong to grade 1 or 2 [24]. In general, these immune-related adverse events are largely associated with inflammatory conditions. For example, adverse events associated with BMS-936558 or pembrolizumab treatments, such as itching and rashes, are the major signs of dermatological inflammation. In order to combat the inflammatory events associated with the use of immune checkpoint inhibitors for cancer treatment, strategies of immunosuppression should therefore be applied in conjunction with immunotherapeutic courses [25]. These strategies may include the systemic treatment of corticosteroid or anti-TNF-α antibody [26], both of which are anti-inflammatory agents.

Overall, to enhance the effectiveness of tumor removal, immunotherapy agents target both the pathways of immune system activation (use of cytokines) and immune system inhibition (immunological checkpoint pathway). However, the likelihood of the development of immune-related adverse events following the utilization of certain immunotherapeutic agents prompts that immunosuppressive therapies may become necessary during the course of immunotherapy.

## 4. Cancer Vaccines in Immunotherapy

In addition to the aforementioned immunotherapy agents, cancer vaccines have also received considerable attention in research on cancer treatment. They normally contain a certain tumor-specific antigen, such as that in the form of peptides derived from a tumor antigen. Therefore, once administered, an immune response can be mounted against the antigen that is only found in tumors. In this way, the immune system would be “trained” to act against the tumors expressing that antigen, thereby enhancing anti-tumor immune responses.

Recently, a number of lung cancer vaccine products have been developed in the form of peptides derived from telomerase and MUC-1, two proteins found to be highly expressed in lung tumors. For example, the administration of GV-1001, a peptide-based vaccine against telomerase, to NSCLC patients was found to immunize these patients against lung tumors by enhancing the immunological memory exhibited by T cells, with minimal adverse side effects [27]. Likewise, L-BLP25, a vaccine comprising a 25-amino-acid peptide derived from MUC-1, was found to increase the time of survival of NSCLC patients after being subcutaneously administered weekly for eight weeks [28]. Overall, cancer vaccines can serve as an alternative strategy in immunotherapy against cancer, through the exploitation of immunological memory in T cells to achieve an enhanced efficiency in the removal of tumors.

## 5. Concluding Remarks

It is clearly evident that immunotherapy has significant advantages over the traditional chemotherapeutic strategies in cancer treatment. Immunotherapy allows a bypass of the problem of tumor resistance to chemotherapeutic drugs. It also offers an opportunity to resolve the problem of immune tolerance of tumors through the use of monoclonal antibodies that block the inhibitory signals for the activation of immune effector cells to eliminate tumors. All these factors contribute to the fact that lung cancer patients receiving immunotherapy can live on average 3.2 months longer than those on a chemotherapy course [22]. Despite the effectiveness of immunotherapy in tumor removal, patients receiving immunotherapy are still likely to suffer from symptoms such as fatigue due to the high energy demand on the immune system to activate and mount an immune response against the tumor. Further, immune-related adverse events are also likely to occur with the use of immune checkpoint inhibitors as immunotherapeutic agents, possibly due to the enhancement of systemic immune responses. In order to reduce the severity of these adverse events, the importance of the strategies for immunosuppression for patients undergoing immunotherapy should not be underestimated. Immunosuppressive therapies that accompany the course of immunotherapy should be in place in an attempt to improve the quality of life of these patients.

## Figures and Tables

**Table 1:** An overview of recent clinical studies demonstrating the benefits of combination therapy over chemotherapy.

Drug Combination and Their Biological Activities	*n*	Main Findings			References
		Clinical Outcome	ChemImm	Chem	
**Immunotherapeutic agent used:** Necitumumab; Biological activity: Block downstream signaling of EGFR that facilitates cell proliferation. **Chemotherapeutic drug used:** (1) Gemcitabine; Biological activity: Prevention of pyrimidine synthesis and hence DNA synthesis. (2) Cisplatin; Biological activity: Induction of DNA alkylation and hence DNA damage.	1093	Median OS	11.5 months (95% CI: 10.4–12.6)	9.9 months (95% CI: 8.9–11.1)	Thatcher *et al.*, 2015 [16]
		Median PFS	5·7 months (95% CI: 5.6–6.0)	5·5 months (95% CI: 4.8–5.6)	
		1-year OS rate	48% (95% CI: 43–52)	43% (95% CI: 39–47)	
		3-month PFS rate	79% (95% CI: 76–83)	73% (95% CI: 68–76)	
		Notes on toxicity profile of combined therapy	Frequency of serious adverse events appear higher, yet comparable, in ChemImm group than Chem group (48% *vs.* 38%).		
**Immunotherapeutic agent used:** Activated T cells and dendritic cells; Biological activity: Removal of metastatic tumors through an immune response. **Chemotherapeutic drug used:** Chemotherapeutic drugs used in the study were not reported.	101	2-year OS rate	93.4% (95% CI: 80.8–97.8)	66.0% (95% CI: 50.4–77.7)	Kimura *et al.*, 2015 [17]
		2-year RFS rate	68.5% (95% CI: 53.2–79.7)	41.4% (95% CI 27.5–54.7)	
		Notes on toxicity profile of combined therapy	44% of patients (22 out of 50) experienced at least one adverse event in the form of shivering, chills and fever.		
**Immunotherapeutic agent used:** Interleukin-2; Biological activity: Stimulation of the growth and differentiation of T cells, for the generation of an immune response against tumors. **Chemotherapeutic drug used:** Gefitinib; Biological activity: Inhibition of EGFR signaling and prevent cell proliferation.	70	Median OS	20.1 months (95% CI: 5.1–35.1)	6.9 months (95% CI: 4.9–8.9)	Bersanelli *et al.*, 2014 [18]
		RR	16.1% (95% CI not reported)	5.1% (95% CI not reported)	
		DCR	41.9% (95% CI not reported)	41.0% (95% CI not reported)	
		Notes on toxicity profile of combined therapy	Grade 2-3 toxicity experienced by patients in the ChemImm group, with fever (46%), fatigue (23%) and arthralgias (13%) being the most common adverse events.		
**Immunotherapeutic agent used:** Cytokine-induced killer cells (CIK); Biological activity: exhibition of cytotoxicity against tumor cells. **Chemotherapeutic drug used:** (1) Navelbine; Biological activity: Induction of microtubule depolymerization, causing the inability of cells to form mitotic spindles and divide. (2) Cisplatin; Biological activity: Induction of DNA alkylation and hence DNA damage.	122	1-year OS rate	57.2% (95% CI not reported)	37.3% (95% CI not reported)	Yang *et al.*, 2013 [19]
		Objective RR	18% (95% CI not reported)	17% (95% CI not reported)	
		DCR	69% (95% CI not reported)	49% (95% CI not reported)	
		Notes on toxicity profile of combined therapy	No severe adverse events reported, despite the occurrence of usual side effects as a result of chemotherapy.		
**Immunotherapeutic agent used:** Cytokine-induced killer cells (CIK); Biological activity: exhibition of cytotoxicity against tumor cells. **Chemotherapeutic drug used:** (1) Paclitaxel; Biological activity: Induction of microtubule depolymerization, causing the inability of cells to form mitotic spindles and divide. (2) Navelbine; Biological activity: Induction of microtubule depolymerization, causing the inability of cells to form mitotic spindles and divide. (3) Gemcitabine; Biological activity: Prevention of pyrimidine synthesis and hence DNA synthesis. (4) Cisplatin; Biological activity: Induction of DNA alkylation and hence DNA damage.	174	Median OS	48 months (95% CI: 29–67)	18 months (95% CI: 11–25)	Li *et al*., 2012 [20]	
		Median PFS	24 months (95% CI: 14–34)	12 months (95% CI: 8–16)		
		3-year OS rate	61% (95% CI: 55–67)	39% (95% CI: 36–42)		
		3-year PFS rate	39% (95% CI: 36–42)	32% (95% CI: 30–34)		
		Notes on toxicity profile of combined therapy	Toxicity profile of the combined therapy was not reported in the study.			
**Immunotherapeutic agent used:** TG-4010; Biological activity: A cancer vaccine against the MUC1 protein, a protein overexpressed in tumors. **Chemotherapeutic drug used:** (1) Gemcitabine; Biological activity: Prevention of pyrimidine synthesis and hence DNA synthesis. (2) Cisplatin; Biological activity: Induction of DNA alkylation and hence DNA damage.	148	Median OS	10.7 months (95% CI: 8.8–18.0)	10.3 months (95% CI: 8.3–12.5)	Quoix *et al.,* 2011 [21]	
		6-month PFS rate	43.2% (95% CI: 33.4–53.5)	35.1% (95% CI: 25.9–45.3)		
		RR	41.9% (95% CI: 30.5–53.9)	28.4% (95% CI: 18.5–40.1)		
		Notes on toxicity profile of combined therapy	Occurrence of more than 1 serious adverse events are comparable between ChemImm and Chem groups (52.1% *vs.* 47.2%).			

ChemImm: the group of patients treated by a combination of immunotherapeutic agent and chemotherapeutic drugs; Chem: the group of patients treated by chemotherapeutic drugs only; CI: confidence interval; DCR: disease control rate; MUC1: Mucin 1; OS: overall survival; PFS: progression-free survival; RFS: recurrence-free survival; RR: relative response.

**Table 2:** Comparison of the clinical outcomes of patients resulting from the use of nivolumab and docetaxel in NSCLC treatment in a clinical study by Borghaei *et al.*, 2015 and Brahmer *et al.*, 2015.

Clinical Outcome	Borghaei *et al.*, 2015 [15] (*n* = 582)		Brahmer *et al.*, 2015 [22] (*n* = 272)	
	Nivolumab	Docetaxel	Nivolumab	Docetaxel
Median OS	12.2 months (95% CI: 9.7–15.1)	9.4 months (95% CI: 8.1–10.7)	9.2 months (95% CI: 7.3–13.3)	6.0 months (95% CI: 5.1–7.3)
HR of death	0.73 (96% CI: 0.59–0.89; *p* = 0.002)		0.59 (95% CI: 0.44–0.79; *p* < 0.001)	
1-year OS rate	51% (95% CI: 45–56)	39% (95% CI: 33–45)	42% (95% CI: 34–50)	24% (95% CI: 17–31)
Median PFS	2.3 months (95% CI: 2.2–3.3)	4.2 months (95% CI: 3.5–4.9)	3.5 months (95% CI: 2.1–4.9)	2.8 months (95% CI: 2.1–3.5)
1-year PFS rate	19% (95% CI: 14–23)	8% (95% CI: 5–12)	21% (95% CI: 14–28)	6% (95% CI: 3–12)
RR	19% (95% CI: 15–24)	12% (95% CI: 9–17)	20% (95% CI: 14–28)	9% (95% CI: 5–15)
Frequency of adverse events	Events of any grade: 69%; Events of grade 3 or 4: 10%	Events of any grade: 88%; Events of grade 3 or 4: 54%	Events of any grade: 58%; Events of grade 3 or 4: 7%	Events of any grade: 86%; Events of grade 3 or 4: 55%

CI: Confidence interval; HR: hazard ratio; OS: Overall survival; PFS: Progression-free survival; RR: Relative response. Note: Biological activity of nivolumab—an immunotherapeutic agent that prevents binding of PD-1 on T cells and PD-1 ligand on tumors, thereby shutting down the inhibitory signal of T cell activation and promoting an immune response against the tumors. *Biological activity of docetaxel*—a chemotherapeutic drug that stabilizes microtubules, preventing microtubule de-polymerization and formation of mitotic spindles, thereby causing the inability of cells to divide.

## References

[1] Ridge C.A., McErlean A.M., Ginsberg M.S. (2013). Epidemiology of lung cancer. Semin. Interv. Radiol..

[2] International Agency for Research on Cancer Globocan 2012: Estimated Cancer Incidence, Mortality and Prevalence Worldwide in 2012. http://globocan.iarc.fr/Pages/fact_sheets_population.aspx.

[3] Chabner B.A., Roberts T.G. (2005). Timeline: Chemotherapy and the war on cancer. Nat. Rev. Cancer..

[4] Ramesh G., Reeves W.B. (2004). Inflammatory cytokines in acute renal failure. Kidney Int. Suppl..

[5] Sharma A., Houshyar R., Bhosale P., Choi J.I., Gulati R., Lall C. (2014). Chemotherapy induced liver abnormalities: An imaging perspective. Clin. Mol. Hepatol..

[6] Liang X.J., Chen C., Zhao Y., Wang P.C. (2010). Circumventing tumor resistance to chemotherapy by nanotechnology. Methods Mol. Biol..

[7] Bidet M., Tomico A., Martin P., Guizouam H., Mollat P., Mus-Veteau I. (2012). The Hedgehog receptor patched functions in multidrug transport and chemotherapy resistance. Mol. Cancer Res..

[8] McCarthy E.F. (2006). The toxins of William B. Coley and the treatment of bone and soft-tissue sarcomas. Iowa Orthop. J..

[9] Turcotte S., Rosenberg S.A. (2011). Immunotherapy for metastatic solid cancers. Adv. Surg..

[10] Eichbaum C., Meyer A.S., Wang N., Bischofs E., Steinborn A., Bruckner T., Brodt P., Sohn C., Eichbaum M.H. (2011). Breast cancer cell-derived cytokines, macrophages and cell adhesion: Implications for metastasis. Anticancer Res..

[11] Jiang T., Zhou C. (2015). The past, present and future of immunotherapy against tumor. Transl. Lung Cancer Res..

[12] Yang S.C., Owen-Schaub L., Mendiguren-Rodriguez A., Grimm E.A., Hong W.K., Roth J.A. (1990). Combination immunotherapy for non-small cell lung cancer. Results with interleukin-2 and tumor necrosis factor-alpha. J. Thorac. Cardiovasc. Surg..

[13] Staveley-O’Carroll K., Sotomayor E., Montgomery J., Borrello I., Hwang L., Fein S., Pardoll D., Levitsky H. (1998). Induction of antigen-specific T cell anergy: An early event in the course of tumor progression. Proc. Natl. Acad. Sci. USA.

[14] Lynch T.J., Bondarenko I., Luft A., Serwatowski P., Barlesi F., Chacko R., Sebastian M., Neal J., Lu H., Cuillerot J.M. (2012). Ipilimumab in combination with paclitaxel and carboplatin as first-line treatment in stage IIIB/IV non-small-cell lung cancer: Results from a randomized, double-blind, multicenter phase II study. J. Clin. Oncol..

[15] Borghaei H., Paz-Ares L., Horn L., Spigel D.R., Steins M., Ready N.E., Chow L.Q., Vokes E.E., Felip E., Holgado E. (2015). Nivolumab *versus* Docetaxel in Advanced Nonsquamous Non-Small-Cell Lung Cancer. N. Engl. J. Med..

[16] Thatcher N., Hirsch F.R., Luft A.V., Szczesna A., Ciuleanu T.E., Dediu M., Ramlau R., Galiulin R.K., Bálint B., Losonczy G. (2015). Necitumumab plus gemcitabine and cisplatin *versus* gemcitabine and cisplatin alone as first-line therapy in patients with stage IV squamous non-small-cell lung cancer (SQUIRE): An open-label, randomised, controlled phase 3 trial. Lancet Oncol..

[17] Kimura H., Matsui Y., Ishikawa A., Nakajima T., Yoshino M., Sakairi Y. (2015). Randomized controlled phase III trial of adjuvant chemo-immunotherapy with activated killer T cells and dendritic cells in patients with resected primary lung cancer. Cancer Immunol. Immunother..

[18] Bersanelli M., Buti S., Camisa R., Brighenti M., Lazzarelli S., Mazza G., Passalacqua R. (2014). Gefitinib plus interleukin-2 in advanced non-small cell lung cancer patients previously treated with chemotherapy. Cancers.

[19] Yang L., Ren B., Li H., Yu J., Cao S., Hao X., Ren X. (2013). Enhanced antitumor effects of DC-activated CIKs to chemotherapy treatment in a single cohort of advanced non-small-cell lung cancer patients. Cancer Immunol. Immunother..

[20] Li R., Wang C., Liu L., Du C., Cao S., Yu J., Wang S.E., Hao X., Ren X., Li H. (2012). Autologous cytokine-induced killer cell immunotherapy in lung cancer: A phase II clinical study. Cancer. Immunol. Immunother..

[21] Quoix E., Ramlau R., Westeel V., Papai Z., Madroszyk A., Riviere A., Koralewski P., Breton J.L., Stoelben E., Braun D. (2011). Therapeutic vaccination with TG4010 and first-line chemotherapy in advanced non-small-cell lung cancer: A controlled phase 2B trial. Lancet Oncol..

[22] Brahmer J., Reckamp K.L., Baas P., Crinò L., Eberhardt W.E., Poddubskaya E., Antonia S., Pluzanski A., Vokes E.E., Holgado E. (2015). Nivolumab *versus* Docetaxel in Advanced Squamous-Cell Non-Small-Cell Lung Cancer. N. Engl. J. Med..

[23] Topalian S.L., Hodi F.S., Brahmer J.R., Gettinger S.N., Smith D.C., McDermott D.F., Powderly J.D., Carvajal R.D., Sosman J.A., Atkins M.B. (2012). Safety, activity, and immune correlates of anti-PD-1 antibody in cancer. N. Engl. J. Med..

[24] Robert C., Ribas A., Wolchok J.D., Hodi F.S., Hamid O., Kefford R., Weber J.S., Joshua A.M., Hwu W.J., Gangadhar T.C. (2014). Anti-programmed-death-receptor-1 treatment with pembrolizumab in ipilimumab-refractory advanced melanoma: A randomised dose-comparison cohort of a phase 1 trial. Lancet.

[25] Villadolid J., Amin A. (2015). Immune checkpoint inhibitors in clinical practice: Update on management of immune-related toxicities. Transl. Lung Cancer Res..

[26] Horvat T.Z., Adel N.G., Dang T.O., Momtaz P., Postow M.A., Callahan M.K., Carvajal R.D., Dickson M.A., D’Angelo S.P., Woo K.M. (2015). Immune-related adverse events, need for systemic immunosuppression, and effects on survival and time to treatment failure in patients with melanoma treated with ipilimumab at memorial sloan kettering cancer center. J. Clin. Oncol..

[27] Brunsvig P.F., Kyte J.A., Kersten C., Sundstrom S., Moller M., Nyakas M., Hansen G.L., Gaudernack G., Aamdal S. (2011). Telomerase peptide vaccination in NSCLC: A phase II trial in stage III patients vaccinated after chemoradiotherapy and an 8-year update on a phase I/II trial. Clin. Cancer Res..

[28] Butts C., Maksymiuk A., Goss G., Soulieres D., Marshall E., Cormier Y., Ellis P.M., Price A., Sawhney R., Beier F. (2011). Updated survival analysis in patients with stage IIIB or IV non-small-cell lung cancer receiving BLP25 liposome vaccine (L-BLP25): Phase IIB randomized, multicenter, open-label trial. J. Cancer Res. Clin. Oncol..

